# Exploration of phylogenetic data using a global sequence analysis method

**DOI:** 10.1186/1471-2148-5-63

**Published:** 2005-11-09

**Authors:** Charles Chapus, Christine Dufraigne, Scott Edwards, Alain Giron, Bernard Fertil, Patrick Deschavanne

**Affiliations:** 1 Equipe de Bioinformatique Génomique et Moléculaire, INSERM U 726, Case 7113, Tour 53-54, 2 place Jussieu, 75005 Paris, France; 2 Inserm U494, 91 bd de l'Hopital 75634 Paris CEDEX 13, France; 3 Dept. of Organismic and Evolutionary Biology, Harvard University, Cambridge, MA 02138 USA; 4 Current address: Dept. of Organismic and Evolutionary Biology, Harvard University, Cambridge, MA 02138 USA

## Abstract

**Background:**

Molecular phylogenetic methods are based on alignments of nucleic or peptidic sequences. The tremendous increase in molecular data permits phylogenetic analyses of very long sequences and of many species, but also requires methods to help manage large datasets.

**Results:**

Here we explore the phylogenetic signal present in molecular data by genomic signatures, defined as the set of frequencies of short oligonucleotides present in DNA sequences. Although violating many of the standard assumptions of traditional phylogenetic analyses – in particular explicit statements of homology inherent in character matrices – the use of the signature does permit the analysis of very long sequences, even those that are unalignable, and is therefore most useful in cases where alignment is questionable. We compare the results obtained by traditional phylogenetic methods to those inferred by the signature method for two genes: RAG1, which is easily alignable, and 18S RNA, where alignments are often ambiguous for some regions. We also apply this method to a multigene data set of 33 genes for 9 bacteria and one archea species as well as to the whole genome of a set of 16 γ-proteobacteria. In addition to delivering phylogenetic results comparable to traditional methods, the comparison of signatures for the sequences involved in the bacterial example identified putative candidates for horizontal gene transfers.

**Conclusion:**

The signature method is therefore a fast tool for exploring phylogenetic data, providing not only a pretreatment for discovering new sequence relationships, but also for identifying cases of sequence evolution that could confound traditional phylogenetic analysis.

## Background

Phylogenetic classifications traditionally rely on phenotypic traits and the paleontological record [1]. As a result of the large amount of DNA sequences now available in the databases, molecular phylogeny has become an essential companion in studying evolutionary relationships among species [2]. As usually practiced, it allows constructing phylogenetic trees based on differences between homologous sequences or genes [3]. A basic and indispensable step in phylogenetic study is alignment of the set of homologous sequences [4]. However, distantly related sequences can be difficult to align and under these conditions, different algorithms often lead to different phylogenetic results [5,6]. There are other problems linked to the use of biological sequences in phylogenetic analysis, including sampling of representative sequences, biological processes such as lateral gene transfer, fusion events and recombination (see Brocchieri *et al *[5] for a review).

New approaches of molecular phylogeny, taking into account new characteristics of sequences, have been recently developed. Such methods include using other aspects of molecular data such as structural properties of proteins [7], the presence and organization of genes along genomes [8-11], occurrence of characteristic patterns [12,13] and the frequencies of short nucleotide or peptide relative abundance [14-18]. These methods contribute to the understanding of species evolution from different points of view, particularly in terms of our understanding of genome evolution. What is intriguing about these methods is that they often yield phylogenetic results comparable to those of traditional methods, frequently employing data sets much larger than traditional phylogenetic analyses. As such, they deserve the attention of those wishing to extract maximal information from comparative genomic data sets.

We expand on a method to characterize DNA sequences: the sequence signature. Sequence signature is defined as the whole set of frequencies of short oligonucleotides (words, until ten nucleotides long currently) of a sequence [19]. The principal characteristics of sequence signature used for phylogenetic studies are species-specificity of sequence signature and conservation of signature in any part of the genome [20] allowing researchers to compare sequences from diverse regions of the genome. It has already been established that distances between species signatures of the same taxonomic group are smaller than between signatures of species belonging to different groups [19,21]. A difference of signatures between two sequences could arise from shifts in the pattern of point substitution, but could also involve interactions among adjacent nucleotides, natural selection, DNA repair processes and conformational constraints (super coiling, nucleosome formation, bend DNA) [22]. A phylogenetic analysis of signatures could therefore reflect underlying genomic changes that shift motif frequencies, thereby yielding higher-order homologies available for phylogenetic analysis. The method has already been used for taxonomic classification of some species groups [23-25]. One advantage of such a method consists mainly in avoiding the alignment step, and can be used on numerous sequences of varying size. In addition, distance matrices, such as those applicable to genomic signatures, generally permit fast building of trees. Perhaps most importantly, genomic signatures provide a means of comparing large-scale patterns in genomes and can help evaluate trends in genome evolution across a phenetic tree. However, no systematic analysis of the reliability of the signature approach has been performed on homologous sequences. It has been demonstrated that long word frequencies describes DNA sequence information more accurately [19,25], but with their much larger number, long words are difficult to apply to short sequences because word frequencies are poorly estimated. Wang *et al*. [25] have also qualitatively analyzed the impact of the choice of the divergence metrics on phylogenetic results. However, no quantitative analyses or simulations have been presented yet on this subject.

In this paper, statistical studies of the ability of a signature approach for reconstructing phylogenies are investigated, specifically in order to determine the optimum word length and the influence of the divergence metric on the results. One of the tests we employ allows us to determine whether the signature distance can be considered tree-like, possessing hierarchical information [26]. Working with homologous, fully alignable sequences, we tested the method on simulated sequences whose true topologies are known and also analyzed two published examples of DNA sequences that propose novel interspecific relationships. Overall we find that there is a strong correspondence between signature trees and those generated by conventional means. As a means of improving large multi-gene studies [27,28], we also propose the use of signatures for rapid, large-scale sequence analysis specifically to detect subsets of genes supporting similar species phylogenies and to identify cases of horizontal transfer. In an analysis of 16 complete γ-proteobacteria genomes, we also illustrate how the signature method can also be used on data sets in which some of gene sequences are missing.

## Results and discussion

### Word length and metrics

**Figure 1:**
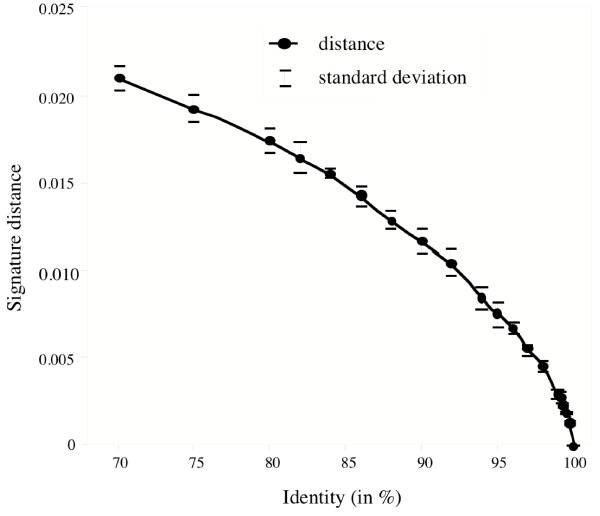
**Signature distance as a function of sequence identity**. Distances obtained from 5 kb sequences. (6 letter-words, Euclidian metric). Each point represents the mean of 100 sequence comparisons. The standard deviation of each point is shown.

In order to determine if the distance between signatures can be relevant in phylogenetic analysis, the signature distances between 2 sequences were plotted as function of their observed sequence identity (Fig 1). We simulated a large set of sequences (100 sequences per point) derived from a reference sequence (random mutations with no homoplasy). The signature of the different sequences – the reference sequence and the whole set of modified sequences – were calculated and compared by Euclidian metric in order to obtain distances to the reference. The same plot was obtained with the χ^2 ^metric. These two metrics lead to quite similar results. The χ^2 ^distance exhibited somewhat more information (steeper slope, better dynamics of the plot) than the Euclidean distance and was consequently used. As shown in figure 1, there is a monotonous increase in distance as the observed sequence identity between sequences increased, suggesting the metrics used to compare signatures may be a valid approach to evaluate differences between sequences.

We then tried to determine how tree-like were the trees inferred by the signature method, and if the distances in our signature matrices reflected tree distances. To do that, we used the distance matrices and the trees of the RAG1 study (see below for a discussion of these results). Various criteria for evaluating treeness, such as arboricity and stress, have been used as proposed by Guénoche and Garetta [26] to answer this question. Considering the three sums involved in the four point condition in quadruples [29], arboricity measures the percentage of quadruples for which the middle sum is closer to the largest one than to the smallest one. Stress corresponds to the square root of the quadratic difference between tree and matrice distances divided by the average distance value. These criteria are numerical and topological. All the criteria have been calculated on the signature-based distance matrices. These distance matrices are obtained using different word lengths (between 1 and 10), because we do not have an *a priori *knowledge of the optimum length.

**Figure 2:**
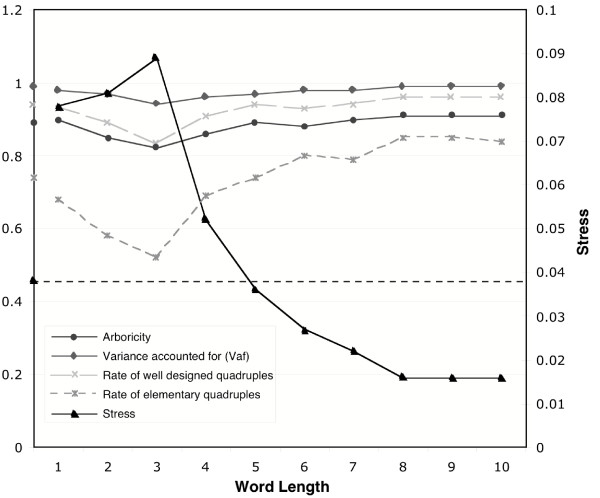
**Dynamics of signature distance matrices**. Distance matrices were obtained from the RAG1 vertebrate study (see below). There are two types of criteria: metric (for example Vaf, stress) and topological (Arboricity, rate of well designed quadruples, rate of elementary quadruples). *Vaf *(variance accounted for): quadratic difference divided by the variance of distance. *Rate of well designed quadruples*: quadruples having the same topology according the two distance matrices; *Rate of elementary quadruples, Arboricity*; see [26]. On the y-axis, the criteria values obtained from the method of distance are plotted. For the stress, this value is indicated also by a dot line.

We found that when word length increases, the arboricity index increases, indicating that the distance improves as a phylogenetic measure (Fig 2). This improvement is clear between 2- and 5-letter words and remains stable for increasing word length. This is in agreement with previous results showing that long words provide better specificity and thus a better taxonomic classification [21]. However, the use of signatures requires that each word occurs frequently enough to provide a good statistical estimation of the true word frequency difference between signatures. The values of the criteria have been also computed for distance matrices of the conventional distance method (Fig 2). From 5-letter words and longer, the criteria from the signature-based distance are better than those of the conventional distance method, especially for the stress criterion. It appears that the different criteria (metric and topological) reached stability and quality for word length around 6-letters. This value of 6 for the word length seems a good trade-off between sequence size and word length and was consequently chosen for additional analyses in this study.

**Figure 3:**
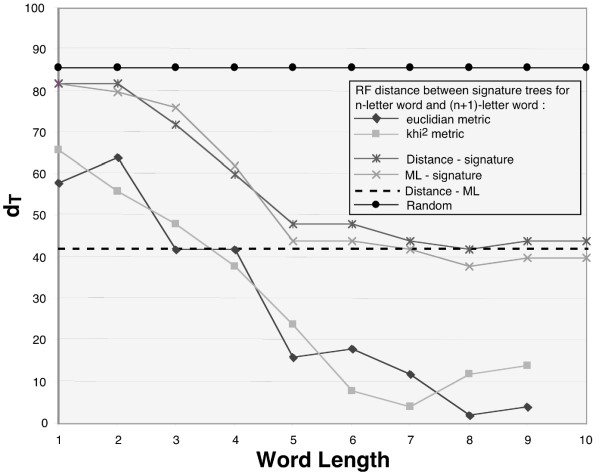
**Robinson-Foulds distance analysis of trees**. The distances were computed from trees of the RAG1 study (see below). For each world length between 1 and 10, a signature tree was computed and compared to the NJ, ML and random trees. For comparison of random trees and signature trees, 100 random trees were built. In this latter case, the *d*_*T *_is approximately 86 (the maximum value possible with this number of species). As a reference, *d*_*T *_between the NJ and ML trees is plotted as dashed line. The *d*_*T *_of the n-/(n+1)-letter word trees was computed for the Euclidean and χ^2 ^metrics.

Are trees for different word lengths converging on a stable tree or is the tree based on each n-letter word different? To compare trees, the tree dissimilarity criterion (*d*_*T*_) of Robinson-Foulds [30-32], a widely used tree comparison metric, was computed for trees based on n- and the (n+1)-letter word for n = 1 to 9. The dissimilarity distance has also been calculated between n-letter word signature trees and trees obtained by ML and distance methods from conventional aligned sequences (Fig. 3).

*d*_*T *_decreases when word length increases (Fig 3), indicating a convergence of the trees towards a stable topology that is reached for 6-letter word whatever the metric used, then for longer word a plateau is observed. The 5- or 6-letter word signature trees are comparable to those obtained by NJ or ML. The *d*_*T *_observed between the signature/NJ or ML trees and those between conventional NJ/ML trees are similar for 5-letter word and higher confirming our choice in 6-letter word for the study.

### Simulation of sequences

We decided to compare signatures trees to known trees using simulated sequences from a known phylogeny. Our simulation tests used a protocol similar to the work of Kumar [33] and Gascuel [34]). 100 phylogenetic trees were chosen randomly among a dataset of the 2000 random trees, proposed by Gascuel to test phylogeny methods [35]. These simulation sets are composed of 24-taxon or 96-taxon trees. For each tree *T*, we used SEQGEN [36] to generate 10 data files with sequences of length 1 kb, 3 kb and 5 kb. These sequences were obtained by simulating the evolution of nucleotides along *T *according to the Kimura two-parameter model with a transition/transversion rate of 2 and a model of site-specific rate heterogeneity following a gamma distribution (with parameter α = 0.75). We obtained for each length of sequence and each number of taxons 1000 data files.

Two reconstruction methods were applied to the simulated sequences: the signature method, using 4, 5 and 6-letter words and the Euclidian and the χ^2 ^metrics, and the distance method using conventional alignments. We used three different evolutionary model: Kimura two-parameter model (same model than the one used to generated the sequences), a simpler model Jukes-Cantor and a more complex HKY85. All the models have been used with a rate of heterogeneity parameter α equal to 0.75. The results are shown in Table 1.

**Table 1:** Simulation results with 1000 trees. The values correspond to the proportion of wrong branches in the inferred trees. Two distance metrics (χ^2 ^and Euclidean) were used with three word lengths. For the distance method, three different evolutionary model have been used : JC, K2P et HKY85.

	24 taxa			96 taxa
sequence length	1 kb	3 kb	5 kb	3 kb

eucl – 4-letter word	17.8	16.3	16.4	20.5
eucl – 5-letter word	13.8	12.0	11.9	16.0
eucl – 6-letter word	12.9	10.7	10.6	14.9

χ^2 ^– 4-letter word	17.6	16.4	16.4	
χ^2 ^– 5-letter word	14.3	12.1	12.0	
χ^2 ^– 6-letter word	14.4	11.4	10.9	

Jukes-Cantor	11.1	6.3	5.2	9.3
Kimura 2-parameter	10.5	6.1	5.0	9.2
HKY 85	10.5	6.1	5.0	9.2

The methods are compared by their ability to infer the "true" tree, i.e. the topology of the tree that has been used to generate the sequences. We used the topological distance *d*_*T *_of Robinson-Foulds between the inferred tree and the true one. The bipartition distance of Robinson-Foulds [30] is equal to the number of bipartition present in one of the two trees and not in the other. The results are presented in term of percentage of misinferred branches. This percentage is equal to the topological distance divided by the maximum number of different bipartition between two trees: 2N-6 where N is the number of taxa.

In both methods, the Neighbor-Joining reconstruction algorithm was used. The differences in the results come principally from the choice of the distance. The Kimura two-parameter can be designed as the "true" distance, because the parameter of the distance are exactly the same as those chosen to generate the sequences. So normally the Kimura distance must be the branch length of the original trees. The fact that the results obtained by the distance method are not perfect can be attributed to the reconstruction algorithm Neighbor Joining (see Gascuel [37]). HKY85 is a model that includes the Kimura 2-parameter (K2P) model, so the result should be the same.

The proportion of wrong branches decreases in the signature method when word length increases (Table 1). At the same time, the longer the sequences, the better the results with the signature method. However, the proportion of correct branches obtained from the signature is not as high as for the distance method. As expected, the results of HKY85 are the same than those of Kimura 2-parameter. The results of the Jukes-Cantor model are similar to those of the signature for 1 k sequences. But for longer sequences, the signature method is less effective than the JC method. The result of K2P can be explained by the fact that the distance method uses exactly the model used to generate the data. This fact also explains why the results of the signature method improve less with the increase of the sequence length than those of the distance method. The fact that, for the moment, no evolutionary model can be design to the signatures limits the estimation of distances between the signatures. An improvement will be to find how the signature evolves with time as function of nucleotide substitution models. Increases in sequence length facilitate estimation of distance by conventional methods, because the substitution model is known. With the signature, 3 kb sequences are sufficient to obtain a representative signature of the species using 6 letter words. As a result, the increase in accuracy between 3 kb and 5 kb is not significant.

Despite the fact that no evolutionary model has been used with the signature, the results obtained from the signature method are reasonable. With 6-letter words, only 10 % of the internal branches are incorrect. It can be compared to the results presented by Gascuel [37]. The results of the signature method are not as good as the distance method, but they are nevertheless rather accurate. In general, the median size of genes is around 1 k. If we use longer sequences, it will be in the case of non-homologous sequences. For long sequences, no conventional method can be applied.

### Vertebrate phylogeny

We used RAG1, a highly conserved gene that produces small distances between sequences to infer the vertebrate phenetic tree [38]. The analysis of the 46 sequences in the dataset had shown that four sequences were complete and the other contained only the conserved core, with length ranging from 1 kb for core sequences to 3 kb for complete ones. This large difference in length induced a bias in the signatures of the four complete sequences, and so in the obtained trees. For comparison with published works [38], we only used the conserved core of RAG1 gene.

**Figure 4:**
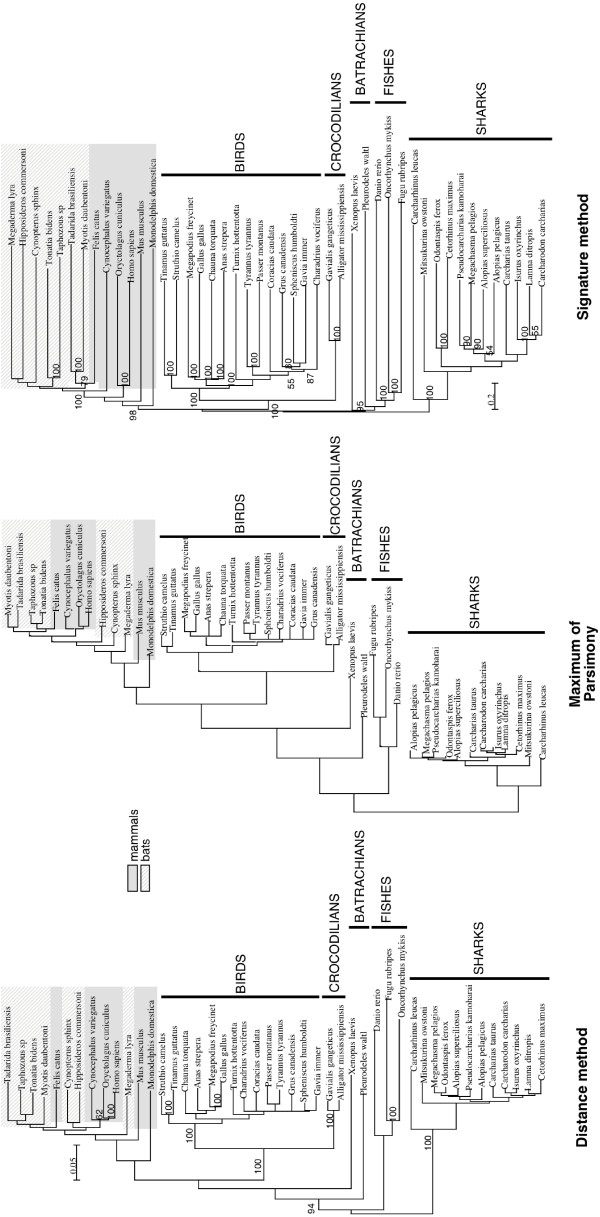
**Phylogeny of vertebrate species**. Three methods were applied to the RAG1 gene from 46 species. *Distance method*: alignment with ClustalW, (Kimura 2-parameter distance), reconstruction by NJ algorithm. *MP*: use of same alignment. PAUP* has been used with default parameters. *Signature method*: 6-letter words – χ^2 ^metric. The tree is inferred by NJ method. The bootstrap coefficients for distance and signature method are indicated.

A phylogenetic tree was inferred for 46 vertebrate sequences by maximum parsimony, distance (nucleic and protein sequences) and the signature method (Fig 4). Trees produced by classical and signature methods show that position of various vertebrate clades (birds, sharks, mammals, fishes, batrachians) is in agreement with paleontological data. The distance tree obtained using protein sequences exhibited some obvious errors: birds presented a stable group but were placed within mammals (data not shown). Moreover, the relationships between species within each taxonomic group are frequently incongruent with other data. The MP method leads to several most parsimonious trees that are summed up into a consensus tree. On the one hand, the major taxonomic groups can be recovered and are placed correctly; on the other hand, positions of species inside these groups are often poorly inferred (for instance, the relationships between mammals are unresolved).

In the signature tree, species are placed within classes in agreement with taxonomy. For example, in the signature analysis, the relationships within birds are congruent with conventional analysis [39]. With regard to mammals, the signature method is the only method that correctly recovers bats as a monophyletic group, with the exception of *Felis catus*. But the cat, *Felis catus*, is misplaced by every method, and so its incorrect placement cannot be attributed to a specific phylogenetic method. Mammal relationships appear much more problematic when analyzed by conventional phylogenetic methods than with the signature method. The polyphyly of tetrapods may be explained by the paucity of batrachian sequences, which could lead to an unreliable position for this clade. The monophyly of taxonomic classes, as well as relationships within each class appear quite robust as measured by bootstrap values.

To determine how strong the phylogenetic signal is present in the signature topology, a congruence analysis of phylogenetic trees [40] can be performed. The topologies obtained by ML, MP (the two best trees), NJ and signature (4- to 6-letter word for the Euclidean and the χ^2 ^metrics) methods, are compared by determining the likelihood of each topology. We establish that the signature trees have a phylogenetic signal similar to the alignment-based ones. The signature trees with long words are more congruent than those using small words. The 6-letter word χ^2 ^signature-tree is congruent with the ML tree and the congruence signature/ML is the same than the congruence NJ/ML (Table 2).

**Table 2:** Difference in log Likelihood. The differences are computed between the ML tree and the other trees.

Tree	Δ-*ln *L
Maximum Likelihood	best

Parsimony	9.38

Distance method	58.95

signature	
χ^2 ^– 4-letter	445.87
χ2 – 5-letter	297.8
χ2 – 6-letter	65.67

Mean random trees	9132.77

### Plant phylogeny

This study, based on an article of Soltis *et al*. [41], used 18S rRNA for 93 plant species whose sequences are available from the "Green Plant Phylogeny Research Coordination Group" . The species can be grouped into nine main clades (Angiosperms (flowering plants), Conifers, Gnetales, Cycads (palm trees), Hornworts, Liverworts, Ferns, Mosses, Lycophytes), with some additional isolated species and an outgroup.

**Figure 5:**
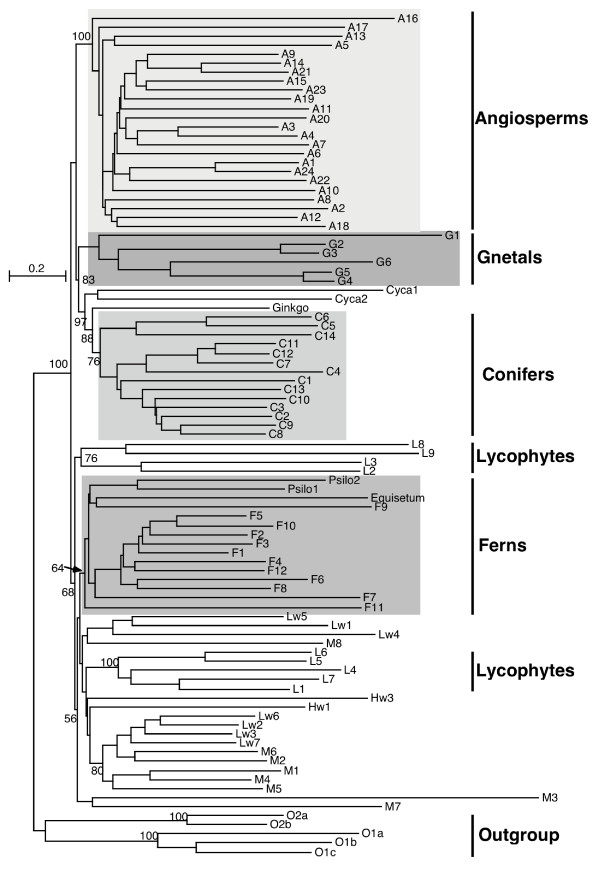
**Phylogenetic tree of plants obtained by comparison of 18S rRNA signatures**. (6-letter words – χ^2 ^metric). The bootstrap coefficients (500 sets) of principal groups are indicated. The species class names are indexed by a code: A – Angiosperm, C – Conifer, G – Gnetale, Cyca – Cycad, F – Fern, M – Moss, L – Lycophyte, Lw – Liverwort, Hw – Hornwort. (see annex for the correspondence code/species).

The signature tree presents significant similarities with the published tree [41]. The angiosperms, conifers, gnetales, cycads and ferns form stable monophyletic groups (high bootstrap coefficients (Fig 5)). The principal result of the article – that the angiosperms are at the root of conifers, gnetales, palm trees and ginkgo (Angiosperm + ((Cycad + Ginkgo) + (Conifern + Gnetale))) – are confirmed by our study and another molecular study [42]. This phylogenetic organization is original as Gnetales are more often linked to Angiosperms by morphological data [43-47] (see Doyle [48] for review).

Recent analyses based on molecular data [49] confirms this result (Soltis [41] and Källersjö [49]). In addition, Equisetum and Psilotaceae are placed with the Ferns. This grouping is found in other studies [50,51] and these species are presented as sister group of Ferns. The sister group relationship of Psilotaceae and Ophioglossaceae is also found [52]. Contrary to the results obtained by Soltis, [41] the ferns are polyphyletic in the signature tree.

The outgroup separates the plants into two groups: the seed plants and the other land plants. To confirm the position of this outgroup, 18S rRNA sequences of *Homo sapiens*, *Saccharomyces cerevisiae *and *Schizosaccharomyces pombe *have been added (Data not shown). The outgroup is still confirmed as well as the tree split. This separation of land/flowering plants, the separation of the Lycophytes and the fact that the moss and liverwort do not form a monophyletic clade have been found also by Soltis when a NJ analysis was performed [41]. Thus, the signature method leads to a similar topology as the NJ method with alignment.

### Multigene trees

Phylogenetic trees carry two types of signal: species evolution and gene evolution. For a variety of reasons, gene trees can be different from the tree of species from which they are sampled [53]. In addition, signals coming from different genes could lead to different inferred phylogenetic relationships between species [54].

In order to deal with this problem, several genes can be used to build a multigene tree [27,28]. The addition of signals coming from various genes can under some conditions reinforce the information on species evolution. In general, the alignment of each gene can be determined, and alignments concatenated prior to tree building. The signature has many properties that facilitate the calculation of multigene tree.

Another problem deals with the selection of genes participating into the multigene tree. In general, several steps of selection occur to eliminate horizontal transferred genes, duplications or those leading to aberrant phylogeny (see [27,28] for an example of these steps). Signatures are an ideal pretreatment tool for identifying horizontally transferred genes [55], and selecting those genes that conform to evolutionary relationships of the species under consideration. Moreover, due to the rapidity of the treatment with the signature, a very large number of genes can be tested at once.

We propose applying the signature method to infer a consensus tree of multiple genes. Two methods are possible. First, assuming that each gene brings the same quantity of information to the phylogeny for each species, an average signature is computed from several genes. The set of average signatures is then analyzed by the signature method. Another approach is to assume that each gene brings a quantity of phylogenetic information that is correlated with its length. In this approach, the sequences are concatenated and signatures are computed on the set of concatenated sequences.

To carry out this study, we used 33 genes originating from ten species (nine Bacteria: *Bacillus subtilis*, *Clostridium perfringens*, *Escherichia coli*, *Lactococcus lactis*, *Neisseria meningitidis*, *Salmonella typhimurium*, *Staphylococcus aureus*, *Vibrio cholerae*, *Xanthomonas axonopodis *and one Archaebacteria:*Archaeoglobus fulgidus *– see Material & Methods).

**Figure 6:**
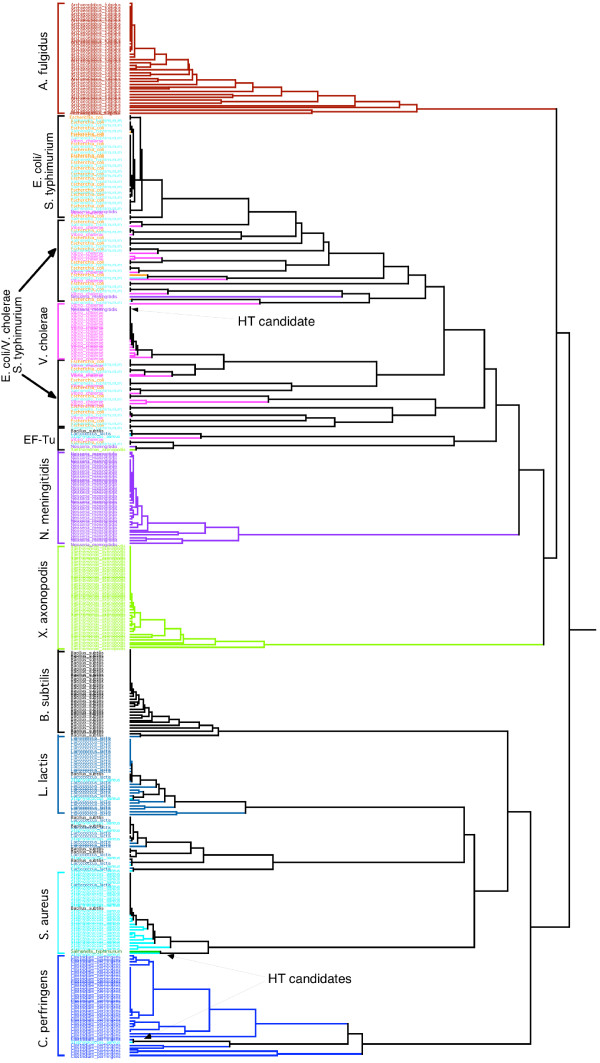
**Hierarchical classification of 393 6-letter word signatures**. The signatures of a given species have the same color code. For each species group, the name of the species is indicated at left. The EF-Tu gene that also forms a stable group is also highlighted. Finally, arrows point out the horizontal transfer (HT) candidates that are discussed in this article.

**Figure 7:**
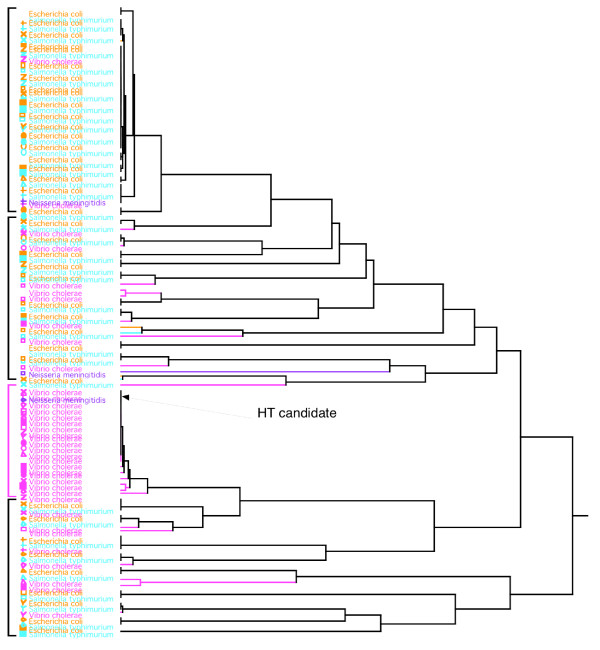
**Detailed view of the hierarchical classification of 393 6-letter word signatures**. A detail focusing on the group with *E. coli*, *S. Typhimurium *and *V. cholerae *is shown. The symbols on the left of the names indicate the genes analyzed.

Because the signature does not rely on statements of homology at the level of individual nucleotides, it is possible to compare signatures from different genes in order to quantify statistical patterns and information content among genes. To determine the relative influence of gene evolution versus species evolution in shaping phylogenetic patterns, all the sequences involved in this study (393 sequences) were compared together by means of a hierarchical classification (Fig 6). The hierarchical classification is an unsupervised method allowing the detection of proximities between complex objects. The main result here is the grouping of gene signatures by species (Fig 6), and the species relationships present some differences with the consensus tree. These relationships are more in agreement with the known topology. *V. cholerae*, *E. coli *and *S. typhimurium *form a stable group, but inside this group, the signatures are grouped by genes (Fig 7). The signature of *V. cholerae *is very close to those of *E. coli*/*S. typhimurium*, as well as in the consensus distance matrix. We clearly face a problem of reconstruction of the Neighbor-Joining algorithm. For *E. coli *and *S. typhimurium*, the differentiation between these two species is quite recent and the homologous genes are very conserved. This leads to an alternate clustering of genes. In the Gram+, the *C. perfringens *signatures are very different to the other and place at the root of the Gram+. This confirms the species specificity of the signature, which was known to be present even in short DNA fragments [20]. The signatures of single genes conserve the characteristics of the species from which they are sampled.

By contrast, an example where gene conservation is very strong is for EF-Tu gene; the signatures of nearly all the species are grouped together at the root of the *V. cholerae*/*E. coli*/*S. typhimurium *group. As it can be observed in the phylogenetic trees (signature and method of distance, results not shown), the *A. fulgidus *and *C. perfringens *copies of the gene are quite different, enough to their species signal to be stronger than the EF-Tu signal.

Some gene signatures cluster with species other than their own in the hierarchical tree. This could result from horizontal gene transfer. For instance, the phosphomannomutase gene of *S. typhimurium *is placed at the root of the *S. aureus *group. In the phosphomannomutase NJ tree and the signature tree, the relationships between the Gram- and the Gram+ bacteria are incongruent with other data and presumably wrong. Despite that, the other phosphomannomutase signatures are correctly assigned to their host species. The misplacement of this gene may indicate a horizontal transfer in *S. typhimurium *from an unknown donor. Two other potential horizontal transfers can be found deep inside species group: the elongation factor 2 signature of *N. meningitidis *and the ornithine carbamoyltransferase signature of *S. aureus *respectively inside the *V. cholerae *group and inside the *C. perfringens *group. In each case the signature is near the signature of the homologous gene of that species. So the gene signal is strong enough to displace the signature inside a different species group. To see if the original sequences are horizontal transfers, we examined two horizontal transfer databases: HGT-DB [56] and HGT Analysis Database [57]. In HGT-DB, the phosphomannomutase sequence of *S. Typhimurium *is tagged as horizontal transfer [56], but not the other two original sequences detected by the hierarchical classification. Thus our novel result suggests original sequences that need to be studied more precisely before being incorporated into a multigene study.

**Figure 8:**
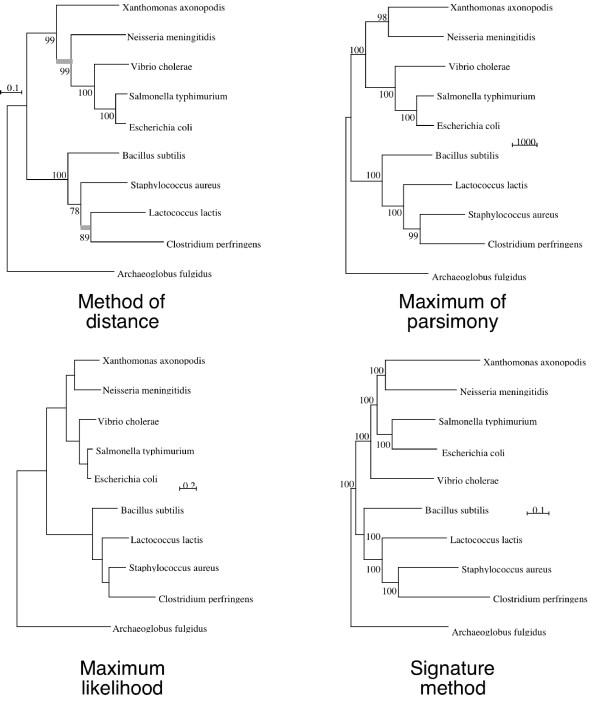
**Consensus trees for ten species**. The four methods shown are the signature (6-letter words – χ^2 ^metric) method, distance method, MP and ML. For each method except ML, the bootstrap coefficients (100 sets) are indicated.

In all the methods, after removal of dubious genes the consensus tree separates the bacteria into the Gram+ and Gram- groups (Fig 8). But for individual genes this topology is seldom obtained. For Gram+ bacteria, the MP and signature methods lead to a (*B. subtilis *+ (*L lactis *+ (*S. aureus *+ *C perfringens*))) grouping, but ML and distance methods place *B. subtilis *deep inside the Gram+ group. For Gram- bacteria, *E. coli *and *S. typhimurium *are always grouped and the majority of the methods (exception maximum of parsimony) place *N meningitidis *and *X axonopodis *together. The principal difference is the place of *V. cholerae *within the Gram-. The ML and MP trees place *V. cholerae *at the root of *E. coli *and *S. typhimurium*. The signature method places *V. cholerae *at the root of Gram- Bacteria.

**Figure 9:**
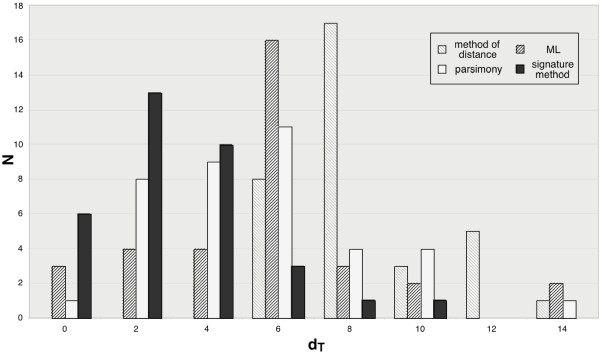
**Dissimilarity distances between the consensus tree and the sets of genes retained**. The *d*_*T *_distances have been computed for the method of distance, ML, MP and signature methods (6-letter word and χ^2 ^metric).

To compare the result of the different studies and to determine the dispersion of the phylogenetic trees, we used the dissimilarity distance between the consensus tree and the whole set of gene trees for distance, MP, ML and the signature method (Fig 9) [32]. The distribution of dissimilarity distances indicates that the signature result is independent of the chosen gene and that each individual gene tree is similar to the consensus tree. In this latter case, the variations mainly arise from the placement of *V. cholerae*, either at the root of Gram- or *E. coli*/*S. typhimurium *clades By contrast, the distance method leads to variable results: no distance tree has a *d*_*T *_lower than 6 when compared to the consensus tree. To a lesser degree, the MP and ML trees exhibit a large dispersion (Table 3). Thus a single gene signature tree is less dissimilar from the consensus tree than a conventional one.

**Table 3:** Statistical analysis of the distribution of dissimilarity distances as a function of method used.

Method	Mean dT	Standard deviation
distance	8.47	2.15
parsimony	5.37	2.98
maximum likelihood	5.65	3.28
signature	3	2.3

The consensus signature method allows us to analyze genes present only for some species. We added 9 genes to the data set (see Materials & Methods), thereby increasing the amount of sequence used to 65 kb per species. The signatures of these genes are amalgamated into the species signatures. The tree obtained (data not shown) exhibits the same topology as the consensus tree obtained with the whole set of genes per species computed previously.

The robustness of the consensus tree topology was assessed by computing 100 bootstrap trees. The bootstrap coefficient was 100% for all branches (Fig 8). Another way to test the robustness of the multigene tree is to vary the number of genes per species included in it, as in a jackknife procedure [58]. In this case, 30, 50, 75 and 90 % of the genes available per species are randomly selected. From the selected genes, an average signature is computed for each species. Distances between these average signatures are used to obtain a signature tree. This procedure is performed 100 times per percentage to yield a bootstrap tree. Results show that the topology of the consensus tree is always the same. However, in some cases the bootstrap coefficients are not maximal (table 4).

**Table 4:** Bootstrap values as function as the number of genes analyzed in the multigene study.

Percentage of used genes	30%	50%	75%	100%
Bootstrap coefficient	100 % except for two clades: – (E. coli + S. typhimurium) = 91 % – (N. meningitides + X. axonopodis) = 96 %	100% for all branches	100% for all branches	100% for all branches

In the individual phylogenetic trees, the variations in topologies are so important whatever the method used (except signature) (Fig 9, Table 3), that they do not allow us to confirm whether these sequences have in fact undergone a horizontal transfer.

### Phylogeny of γ-proteobacteria

**Figure 10:**
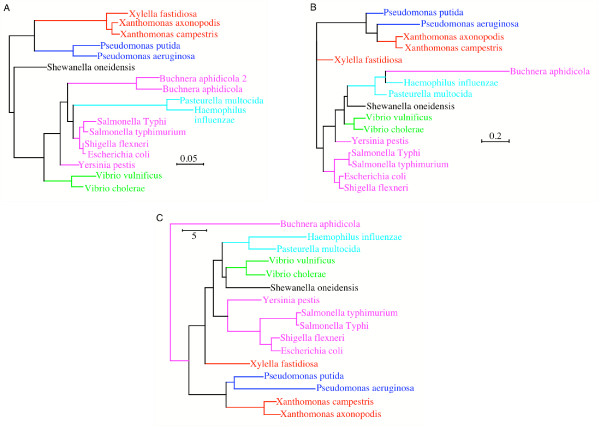
**A- Tree of γ-proteobacteria obtained from the MP method for the 16S rRNA sequences**. Each color corresponds to a taxonomic group. **B- Tree of γ-proteobacteria obtained from non-corrected signatures **(6-letter word signatures and City Block metric). Each color corresponds to a taxonomic group. **C- Tree of γ-proteobacteria obtained from the signatures corrected by a zero order Markov model **signatures (6-letter word signatures and City Block metric). Each color corresponds to a taxonomic group.

We have shown that using signatures and comparing non-homologous sequences such as are found in complete genomes made it possible to determine the relationship between species. To extend the results obtained with 10 prokaryotes genomes, we explore phylogenetic relationship of a well-studied taxonomic group: the γ-proteobacteria [28]. We selected 16 species whose complete genomes are available. These species can be classified in 6 taxonomic groups (Table 5). Pride *et al*. [15] used corrected signatures to infer phylogenetic trees. The signatures were corrected by zero order Markov model to normalize the base composition of the different species. Pride *et al*. [15] determined that this correction permits to obtain a signature tree the most congruent with the 16S rRNA tree. In order to compare the results to a reference, the 16S rRNA sequences have been used to infer a tree by the ML method (Fig 10A). A comparison of trees using signatures corrected and not corrected for base compositional biases is shown in Figures 10B and 10C.

**Table 5:** Species names and taxonomic groups of γ-proteobacteria.

Species name	Taxonomic group
*Shewanella oneidensis*	Alteromonadale
*Buchnera aphidicola*	Enterobacteriale
*Escherichia coli*	Enterobacteriale
*Salmonella typhi*	Enterobacteriale
*Salmonella typhimurium*	Enterobacteriale
*Shigella flexneri*	Enterobacteriale
*Yersinia pestis*	Enterobacteriale
*Haemophilus influenzae*	Pasteurellale
*Pasteurella multocida*	Pasteurellale
*Pseudomonas aeruginosa*	Pseudomonaceae
*Pseudomonas putida*	Pseudomonaceae
*Vibrio cholerae*	Vibrionale
*Vibrio vulnificus*	Vibrionale
*Xanthomonas axonopodis*	Xanthomonadale
*Xanthomonas campestris*	Xanthomonadale
*Xylella fastidiosa*	Xanthomonadale

The 16S rRNA tree permits the establishment of reference relationships between the γ-proteobacteria. Some taxonomic groups are recovered: Xanthomonadales, Pseudonomaces as well as Pasteurelles. The tree groups Xanthomonadales and Pseudodomaces, and places *B aphidicola *close to Pasteurellale but with a long branch. This long branch can explain the incongruent placement of Pasteurelles in Enterobacteria for the ML tree (the phenomenon of long branch attraction [59]).

The tree calculated using the base compositionally-corrected signatures of complete genomes is more in agreement with the 16S rRNA tree. A group of Enterobacteria similar to that found in the 16S rRNA signature tree was obtained. However the monophyly of Xanthomonadales is not recovered in any of the trees obtained from signatures of complete genomes. *X fastidiosa *is placed at the root of the group (Xanthomonadale + Pseudomaceae). Another difference between trees for complete genomes and those of the 16S RNA is a grouping of Pasteurellales, Vibrionales and *S. oneidensis *found in the signature tree.

In the complete genome trees, *B aphidicola *appears misplaced. It is always positioned apart from the Enterobacterial clade, despite its belonging to this group taxonomically. An analysis of genome signatures of *B aphidicola *revealed that this species exhibits a very different signature from those of the other γ-proteobacteria (result not shown). This result is not due to a bias in signature method arising from the size of *B aphidicola *genome, because a tree obtained by randomly selecting the same sequence length in the 15 other genomes (650 kb) leads to the same topology (result not shown). We suggest that the source of this anomoly is that *B aphidicola *is a symbiotic bacteria, andhas a very small genome (650 KB) compared with those of the other γ-proteobacteria (4 to 5 Mb). This genome reduction arises from its parasitic lifestyle and is the result of many independent losses of genes and genomic segments. *B aphidicola *experienced very strong evolutionary pressures that led to a profound shift in its signature, and also transferred numerous genes to its host [60]. Such symbiotic species are known to be difficult to place phylogenetically [61]. *B. aphidicola *also has a strong compositional bias (the genome of is nearly 75% AT rich). The other γ-proteobacteria are more GC rich. These problems appear when using whole genomes to infer a tree and are bypassed when using conserved genes or a selection of genes sharing a common history [28].

We used the method of Dufraigne *et al*. [55] to detect in the *B aphidicola *genome sequences that may have arisen by horizontal transfer. We divided the entire genome into 5 kb sequence windows. For each window, a 4-letter word signature was computed. The method developed by Dufraigne *et al*. allows us to detect which sequences have original signatures such as would be found in cases of horizontal tranfer. We removed this original sequences from the genome and a new 6-letter word signature was computed. The tree obtained is exactly the same as the base compositionally-corrected tree (Fig 10C).

## Conclusion

In this paper we have illustrated the exploration of phylogenetic data with a global sequence analysis method, the signature method. Using a variety of genes, this method yields tree topologies similar to those obtained using traditional phylogenetic approaches. The results presented here suggest that trees obtained by this method could be used as an exploratory step in phylogenetic studies. The signature method can deliver a quick overview of phylogenetic relationships between species in data sets that can be challenging or time consuming for traditional alignment and phylogenetic analysis. As our simulations showed, the signature method sometimes yields phylogenies that are less accurate than those produced by conventional analyses, but this arises mainly from the fact that no evolutionary model is known for word frequencies comprising genomic signatures. The signature tree can be used as fast pretreatment in conjunction with classical methods such as ML. We also demonstrated that the signature distances are tree-like, reflect tree distances and that in the case of short sequences such as frequently assembled in studies of homologous sequences, the optimal word length seems to be 6. This length represents a trade-off between long words that represent more accurately the DNA sequences [21,25] and the size of the sequences.

The signature method is particularly useful as a first step in data exploration. The speed of the analysis permits detection of either misplacement of particular species, in some cases due to local composition fluctuations (horizontal transfer), or unexpected groupings of species that can be scrutinized further by biological means or conventional phylogenetic study. Thus, the signature method easily permits the researcher to use long and/or numerous genes in a study. When using numerous species, their phylogenetic proximities can be analyzed using their signatures by conventional statistical methods and the set of species split into subgroups. This method is also useful in combining information from different genes. The signature method permits the averaging of a great number of genes of any length to obtain a consensus and a unique signature per species and thereby take into account a great number of evolutionary events. The signature method does not rely on homology of DNA sites to compare sequences and it is possible to compare non-homologous sequences to infer a phylogenetic tree. Thus, many genes not present in every species can be added to this tree, giving more confidence in the species tree. This approach was already applied to birds [24], bacterial [15-18,21,62] or mitochondrial [25] phylogenetic studies. In contrast to conventional methods, the signature method utilizes information present in the sequences that may not be analyzable with conventional alignments, such as additional sequences at the beginning or the end of alignments.

For studies of complete genomes, detection of horizontal transfer using signatures, such as proposed by Dufraigne *et al*. [55], permits removal of sequences that will compromise phylogenetic analysis. Finally, signatures allow the rapid detection of horizontally transferred genes or simply misplaced genes that require additional attention via hierarchical clustering or other statistical classification methods.

## Methods

### Sequence signature

Sequence signature can be computed easily and very quickly thanks to an algorithm -the "Chaos game representation" (CGR)-, (about 1 Mb per second on a laptop computer) [63]. The signature can be displayed as an image, where each pixel represents a word and the darkness of the pixel increases with the frequency of the word in the sequence.

### DNA sequences

We selected two genes to compare signature analysis of two different clades with results from the literature. These genes are long enough to get a significant signature and address the phylogeny of vertebrates and plants including a large number of species. The recombination activation gene RAG1 is used for inferring the phenetic tree of 46 species of vertebrates. Ribosomal RNA sequence analysis is the *de facto *standard for phylogenetic reconstruction. Here we use ribosomal 18S RNA to analyze 93 plant species. Finally, 42 genes, accounting for more than 50 kb of sequence, are used for a multigene study (see Annex), including nine Bacteria and one Archaea. To select the 42 genes, we utilized the SYSTERS database [64]. For all the selected species, the database returned 119 orthologous protein families shared by the whole set of species. These families were filtered by size of the corresponding DNA sequences (retained families contain sequences with mean lengths > 1 kb). From these, 33 complete sets and 9 partial sets of genes were obtained. The selected genes belong mainly to amino acid, nucleotide and protein synthesis and DNA metabolism families. All the sequences were extracted from GenBank or Genome Information Broker [65]. The complete genomes of 16 γ-proteobacteria were gathered from GenBank (see appendix). Simulated sequences from a known phylogeny were found on Gascuel's website [35].

### Phylogenetic analysis and signature method

Two distance metrics (Euclidean and ?2) were used to quantify the differences between signatures. Other metrics (Manhattan, Mahalanobis, Correlation and Cosine) were investigated as well; these methods rarely performed better than our two focal methods, and often performed worse, so we do not consider them further. Distance matrices were obtained via the Euclidean and ?2 metrics. We used these matrices to infer trees with the Neighbor-Joining (NJ) reconstruction algorithm implemented in the PHYLIP package [66]. In order to estimate the robustness of the tree topology, we simulated by bootstrap [67] a whole new set of signatures from the initial set of motif frequencies, sampling with replacement (in general, 100 bootstrap trees were computed). Each dataset contains the same individuals from the initial data and N new variables (words) randomly drawn in order to replace the N variables from the initial set [67]. For each set of sequences, the phylogenetic tree was inferred and a consensus tree was calculated from each bootstrap replicate. Besides the signature method, three commonly used methods [3] were used to analyze aligned sequences from the same data sets: Neighbor-Joining (NJ) [68], maximum parsimony (MP) [69] and maximum of likelihood (ML) [2]. All three methods were implemented using the PAUP* [70] and PHYLIP packages. Alignments were obtained with ClustalW (default parameters)[4] and were similar to those used in their respective sources. For the different conventional methods, we have used the HKY85 model of sequence evolution, and gaps were treated as missing data in the MP analysis. For ML analyses, a gamma distribution of rate heterogeneity with simultaneous parameter estimation was used.

## Appendix

### Species annotation for the 18S rRNA sequences of plants

A1: *Asarum canadense*; A2: *Sparganium eurycarpum*; A3: *Tetracentron sinense*; A4: *Trochodendron aralioides*; A5: *Austrobaileya scandens*; A6: *Sassafras albidum*; A7: *Akebia quinata*; A8: *Amborella trichopoda*; A9: *Camptotheca acuminata*; A10: *Gossypium hirsutum*; A11: *Celtis yunnanensis*; A12: *Canna coccinea*; A13: *Ceratophyllum demersum*; A14: *Dipsacus sp*; A15: *Liquidambar styraciflua*; A16: *Zea mays*; A17: *Nymphaea tuberosa*; A18: *Oncidium excavatum*; A19: *Phytolacca americana*; A20: *Pisum sativum*; A21: *Symphoricarpos albus*; A22: *Saururus cernuus*; A23: *Saxifraga integrifolia*; A24: *Saruma henryi*; C1: *Araucaria excelsa*; C2: *Cephalotaxus wilsoniana*; C3: *Juniperus chinensis*; C4: *Phyllocladus trichomonoides*; C5: *Pinus elliottii*; C6: *Pinus luchuensis*; C7: *Dacrycarpus imbricatus*; C8: *Amentotaxus formosana*; C9: *Torreya nucifera*; C10: *Taiwania cryptomerioides*; C11: *Podocarpus costalis*; C12: *Nageia nagi*; C13: *Taxus chinensis var. mairei*; C14: *Abies lasiocarpa*; Cyca1: *Cycas taitungensis*; Cyca2: *Zamia pumila*; Equisetum: *Equisetum hyemale*; F1: *Adiantum raddianum*; F2: *Blechnum occidentale*; F3: *Dicksonia antarctica*; F4: *Dicranopteris linearis*; F5: *Hypolepis muelleri*; F6: *Lonchitis hirsuta*; F7: *Osmunda cinnamomea*; F8: *Odontosoria chinensis*; F9: *Ophioglossum petiolatum*; F10: *Pteridium aquilinum*; F11: *Salvinia natans*; F12: *Vandenboschia davallioides*; G1: *Welwitschia mirabilis*; G2: *Ephedra sinica*; G3: *Ephedra torreyana*; G4: *Gnetum nodiflorum*; G5: *Gnetum urens*; G6: *Gnetum gnemon*; Ginkgo: *Ginkgo biloba*; Hw1: *Anthoceros agrestis*; Hw2: *Notothylas breutelii*; Hw3: *Phaeoceros laevis*; L1: *Huperzia lucidula*; L2: *Isoetes durieui*; L3: *Isoetes engelmannii*; L4: *Lycopodiella inundata*; L5: *Huperzia phlegmaria*; L6: *Huperzia taxifolia*; L7: *Lycopodium tristachyum*; L8: *Selaginella umbrosa*; L9: *Selaginella vogelii*; Lw1: *Marchantia polymorpha*; Lw2: *Fossombronia pusilla*; Lw3: *Pellia epiphylla*; Lw4: *Reboulia hemisphaerica*; Lw5: *Sphaerocarpos donnelli*; Lw6: *Scapania nemorea*; Lw7: *Riccardia pinguis*; M1: *Physcomitrella patens*; M2: *Atrichum undulatum*; M3: *Eurhynchium hians*; M4: *Funaria hygrometrica*; M5: *Leptobryum pyriforme*; M6: *Polytrichum formosum*; M7: *Physcomitrium pyriforme*; M8: *Sphagnum cuspidatum*; O1a: *Chara australis*; O1b: *Chara connivens*; O1c: *Chara foetida*; O2a: *Nitella flexilis*; O2b: *Nitella sp*; Psilo1: *Psilotum nudum*; Psilo2: *Tmesipteris tannensi*.

**Table 6:** Genes used in multigene study:

1/ whole set of species:	
Adenylosuccinate lyase	1.3 kb
Adenylosuccinate synthetase	1.3 kb
Alanyl-tRNA synthetase	2.6 kb
Argininosuccinate synthase	1.3 kb
Argininosuccinate lyase	1.4 kb
Arginyl-tRNA synthetase	1.7 kb
Aspartate aminotransferase	1.2 kb
Aspartyl-tRNA synthetase	1.8 kb
Carbamyl-phosphate synthase	3.2 kb
Cell division protein ftsZ	1.2 kb
Chorismate synthase	1.1 kb
CTP synthase	1.6 kb
DNA-directed RNA polymerase	2.6 kb
DNA topoisomerase I	2.0 kb
Elongation factor 2	2.1 kb
Enolase	1.3 kb
5-enolpyruvylshikimate-3-phosphate synthetase	1.3 kb
Glutamine synthetase	1.5 kb
Leucyl-tRNA synthetase	2.8 kb
Methionyl-tRNA synthetase	2.1 kb
Ornithine carbamoyltransferase	1.0 kb
Pantothenate metabolism flavoprotein	1.2 kb
D-3-phosphoglycerate dehydrogenase	1.2 kb
Phosphoglycerate kinase	1.2 kb
Phosphomannomutase	1.3 kb
Phosphoribosylformylglycinamidine synthase II	3.8 kb
Queuine tRNA-ribosyltransferase	1.1 kb
Ribonucleotide reductase	2.3 kb
Serine hydroxymethyltransferase	1.2 kb
Thermosome alpha subunit	1.6 kb
Threonyl-tRNA synthetase	2.0 kb
Translation elongation factor EF-Tu	1.3 kb
Valyl-tRNA synthetase	2.6 kb
Total length = 57.2 kb	
2/ partial set of species:	
Acetolactate synthase large subunit	1.7 kb
Cysteinyl-tRNA synthetase	1.4 kb
Galactosyltransferase	1.1 kb
GTP cyclohydrolase II	1.1 kb
Histidine kinase	2.0 kb
Phosphoenolpyruvate synthase	2.4 kb
dTDP-glucose 4,6-dehydratase	1.1 kb
Tryptophan synthase subunit beta	1.2 kb
X-pro aminopeptidase	1.3 kb

## Authors' contributions

CC and DP conceived the study, drew the figures and wrote the first draft of the manuscript. CC was the main contributor of the bioinformatic analysis. CD participated in the bioinformatic study. SE participated in the method design and drafted the manuscript. AG and BF drafted the manuscript. All authors read and approved the final manuscript.
